# Genetically predicted N-methylhydroxyproline levels mediate the association between naive CD8+ T cells and allergic rhinitis: a mediation Mendelian randomization study

**DOI:** 10.3389/fimmu.2024.1396246

**Published:** 2024-05-23

**Authors:** Zhengjie Chen, Ying Suo, Xintao Du, Xiaoyun Zhao

**Affiliations:** ^1^ Clinical School of Thoracic, Tianjin Medical University, Tianjin, China; ^2^ Department of Respiratory and Critical Care Medicine, Tianjin Chest Hospital, Tianjin, China; ^3^ Department of Respiratory and Critical Care Medicine, Chest Hospital of Tianjin University, Tianjin, China; ^4^ DeepinBreath Union Laboratory, Tianjin Chest Hospital, Tianjin, China

**Keywords:** allergic rhinitis, immunity, metabolites, causal inference, Mendelian randomization, mediation

## Abstract

**Background:**

Allergic rhinitis (AR), a prevalent chronic inflammatory condition triggered by immunoglobulin E (IgE), involves pivotal roles of immune and metabolic factors in its onset and progression. However, the intricacies and uncertainties in clinical research render current investigations into their interplay somewhat inadequate.

**Objective:**

To elucidate the causal relationships between immune cells, metabolites, and AR, we conducted a mediation Mendelian randomization (MR) analysis.

**Methods:**

Leveraging comprehensive publicly accessible summary-level data from genome-wide association studies (GWAS), this study employed the two-sample MR research method to investigate causal relationships among 731 immune cell phenotypes, 1400 metabolite levels, and AR. Additionally, employing the mediation MR approach, the study analyzed potential mediated effect of metabolites in the relationships between immune cells and AR. Various sensitivity analysis methods were systematically employed to ensure the robustness of the results.

**Results:**

Following false discovery rate (FDR) correction, we identified three immune cell phenotypes as protective factors for AR: Naive CD8br %CD8br (odds ratio (OR): 0.978, 95% CI = 0.966–0.990, P = 4.5×10^–4^), CD3 on CD39+ activated Treg (OR: 0.947, 95% CI = 0.923–0.972, P = 3×10^–5^), HVEM on CD45RA- CD4+ (OR: 0.967, 95% CI = 0.948–0.986, P = 4×10^–5^). Additionally, three metabolite levels were identified as risk factors for AR: N-methylhydroxyproline levels (OR: 1.219, 95% CI = 1.104–1.346, P = 9×10^–5^), N-acetylneuraminate levels (OR: 1.133, 95% CI = 1.061–1.211, P = 1.7×10^–4^), 1-stearoyl-2-arachidonoyl-gpc (18:0/20:4) levels (OR: 1.058, 95% CI = 1.029–1.087, P = 5×10^–5^). Mediation MR analysis indicated a causal relationship between Naive CD8br %CD8br and N-methylhydroxyproline levels, acting as a protective factor (OR: 0.971, 95% CI = 0.950–0.992, P = 8.31×10^–3^). The mediated effect was -0.00574, accounting for 26.1% of the total effect, with a direct effect of -0.01626. Naive CD8+ T cells exert a protective effect on AR by reducing N-methylhydroxyproline levels.

**Conclusion:**

Our study, delving into genetic information, has substantiated the intricate connection between immune cell phenotypes and metabolite levels with AR. This reveals a potential pathway to prevent the onset of AR, providing guiding directions for future clinical investigations.

## 1 Introduction

Allergic rhinitis (AR), a prevalent chronic inflammatory condition triggered by immunoglobulin E (IgE) in response to inhaled allergens (1), manifests widely across age groups, with estimated prevalence rates in European populations ranging from 17% to 28.5% (2, 3). Moreover, its incidence has steadily increased in recent years (4–6), imposing substantial health and psychological burdens due to its seasonal and recurrent nature.

Long-term exposure to allergens may compromise the airway epithelial cells’ barrier function, inducing inflammatory responses in the airways and triggering the onset of AR (7). Elevated levels of group 2 innate lymphoid cells (ILC2s) have been observed in AR patients (8), while myeloid dendritic cells (mDCs) are capable of secreting IL-33 to activate ILC2s via the IL-33/ST2 pathway (9). Additionally, IL-25, IL-33, and thymic stromal lymphopoietin (TSLP) can individually or collectively stimulate ILC2s to produce IL-4, IL-5, and IL-13, initiating immune responses that may manifest as inflammation and allergic symptoms in nasal mucosal tissues, such as congestion and rhinorrhea (10). Moreover, memory-type pathogenic Th2 cells, detected in the peripheral blood of symptomatic AR patients, exhibit heightened IL-5 and IL-9 levels compared to conventional Th2 cells (11), potentially correlating with clinical symptoms (12). Furthermore, a circulating memory B cell subset expressing high levels of CD23 correlates with allergen-specific IgE levels and symptom severity in AR patients (13). Dendritic cells, as primary antigen-presenting cells, uptake allergens and present them to CD4+ T cells, provoking allergen-induced inflammation (14, 15). Thus, allergic rhinitis, characterized as a chronic hypersensitivity disorder, involves diverse immune cells and cytokine-mediated responses in its progression (16). Elucidating these possible pathways is crucial for advancing AR treatment.

With the continuous advancement of high-throughput sequencing technology, there has been a more profound exploration of immune cell functions. Immune cells undergo intricate genetic regulatory processes, and a deeper understanding of these mechanisms holds the potential to unveil the mysteries of the immune system. Identifying and targeting specific points can enhance drug design and development, offering novel perspectives for the treatment and prevention of AR (17).

Allergen Immunotherapy (AIT) induces allergen tolerance by disrupting immune pathogenic mechanisms of allergic reactions and is currently considered an effective treatment for AR (18). Retrospective cohort studies have demonstrated that AIT can reduce the incidence of asthma attacks and pneumonia in AR patients (19). Over the years, various treatment forms have been developed for different patients, including Subcutaneous Immunotherapy (SCIT), Sublingual Immunotherapy (SLIT), and Intralymphatic Immunotherapy (ILIT) (20). However, owing to the complexity and uncertainty of clinical studies, our understanding of the interaction between the immune system and AR remains somewhat limited. Convincing evidence regarding the regulatory effects of AIT on immune cells in the human body is lacking due to variations in race, region, and individual factors. This may be attributed to insufficient sample sizes in real-world studies, divergent study designs, and challenges in eliminating confounding factors. Given that AIT is an individualized treatment approach based on the clinical and immunological characteristics of patients, identifying and validating biomarkers as treatment targets for AR has become a crucial direction in current AIT research (21).

With the disclosure of phenotype-related genetic information, a novel scientific research method has emerged—Mendelian randomization (MR). In recent years, MR has gained popularity as a research approach that employs genetic information loci strongly correlated with exposure factors as instrumental variables (IVs) to infer causal relationships between exposure factors and study outcomes. The method is grounded in Mendel’s laws of inheritance, where alleles are randomly allocated to offspring during meiosis, largely mitigating the influence of confounding factors and reverse causation (22). Immune cells play a pivotal role in the onset and progression of AR. However, current research has only addressed a fraction of them. Due to the intricate biological mechanisms of the immune system, the precise roles of many immune cells remain incompletely understood. Hence, we conducted a comprehensive causal association analysis between immune cell phenotypes and AR through MR, leveraging extensive genome-wide association studies (GWAS) summary-level data of immune cell phenotypes. The goal is to reveal the intricate relationship between immune cell phenotypes and AR.

Employing a two-sample MR approach, we conducted a study investigating the causal relationships involving 731 immune cell phenotypes and AR. Additionally, recognizing the involvement of metabolite levels in the onset and progression of AR, we further evaluated the causal associations between 1400 blood metabolite levels and AR. As part of the MR research, mediation MR can assess the effects of intermediate factors between exposure and outcome, offering insights into whether exposure factors exert their effects through these intermediaries (23). Consequently, we also scrutinized the mediated effect of blood metabolite levels in the relationship between immune cell phenotypes and AR. It is imperative to emphasize that for immune cell phenotypes with a causal relationship to AR, there should be no reverse causation. Accordingly, for positive results, we conducted an MR analysis of AR and immune cell phenotypes to eliminate reverse causation.

## 2 Materials and methods

### 2.1 MR assumptions

It is essential to clarify that MR relies on three fundamental assumptions: (1) The assumption of association, suggesting a robust correlation between the selected genetic variations serving as IVs and the exposure factors. (2) The assumption of independence, stating that genetic variations are unrelated to confounding factors. (3) The assumption of exclusivity, proposing that genetic variations can solely influence the outcome through exposure (24). The study adheres to the STROBE-MR statement (25). Furthermore, the GWAS summary-level data used in this research are publicly accessible, and the ethics committees of each institutional review board granted written informed consent from all participants in individual studies. Since this study involves a secondary analysis of previously published data, ethical approval is considered not required. Significantly, there was no observed overlap, as samples of AR, immune cells, and metabolites originated from distinct consortia.

### 2.2 GWAS data sources for AR

Summary-level data regarding AR were extracted from a comprehensive European population-wide GWAS conducted within the FinnGen project in Finland (26). We specifically selected the most recent R10 version summary data on AR, which encompasses 12,240 cases and 392,069 controls. The diagnosis of AR relied on the International Classification of Diseases codes, specifically ICD-10 (J30.1-J30.4) and ICD-9 (477). Additional details can be accessed at (https://www.finngen.fi/en/access_results).

### 2.3 Immune cells GWAS data sources

A genetic variation analysis was conducted on 731 immune cell phenotypes within a cohort of 3,757 Sardinian individuals. This analysis covered 118 absolute cell counts (AC), 389 median fluorescence intensities (MFI) representing surface antigen levels, 32 morphological features (MP), and 192 relative cell counts (RC) (17). The GWAS data for these phenotypes can be accessed on the IEU Open GWAS project (https://gwas.mrcieu.ac.uk) through identifiers (ebi-a-GCST0001391 to ebi-a-GCST90002121).

### 2.4 Metabolites GWAS data sources

In another investigation with 8,299 participants from the Canadian Longitudinal Study on Aging (CLSA) cohort, researchers performed a GWAS on 1,091 blood metabolites and 309 metabolite ratios (27). This study yields vital insights into elucidating the genetic architecture of metabolites. The summary data for 1,400 metabolite levels are available in the GWAS Catalog (https://www.ebi.ac.uk/gwas) under identifiers (GCST90199621 to GCST90201020).

### 2.5 Selection of IVs

In MR analysis, we employed single nucleotide polymorphisms (SNPs) strongly correlated with the exposure as IVs. Recent studies revealed a limited number of SNPs strongly associated with immune cell phenotypes and blood metabolite levels using a genome-wide significance threshold of P < 5×10^–8^. Therefore, drawing on prior research, we adopted a less stringent threshold (P < 1×10^–5^) for SNPs selection (28, 29). Linkage disequilibrium was eliminated within a genetic distance of kb = 10,000 (correlation coefficient r^2^ < 0.001) to ensure SNP independence. In reverse MR analysis, SNPs in AR summary data were chosen with a threshold of P < 5×10^–8^, and the same method (kb = 10,000, r^2^ < 0.001) was applied to eliminate linkage disequilibrium (30). Palindrome SNPs, having identical alleles on the forward and reverse strands, were excluded due to the ambiguity in determining the effect allele strand. Finally, for each SNP meeting the specified criteria, R^2^ and F-statistic were calculated, excluding SNPs with F-statistic < 10 as weak IVs, indicating a weak correlation between genetic variation and exposure (31).

### 2.6 Statistical analysis

Data analyses were conducted using MRPRESSO (version 1.0) and TwoSampleMR (version 0.5.6) packages in R software (version 4.3.1). Employing five MR analysis methods, with the Inverse Variance Weighted (IVW) method as the primary one, we assessed the causal relationship between exposure and outcome. To ensure robustness, we conducted sensitivity analyses using methods with different assumptions about horizontal pleiotropy, such as MR-Egger and Mendelian Randomization Pleiotropy Residual Sum and Outlier (MR-PRESSO). MR-Egger analysis evaluated instrumental variable pleiotropy, with a non-zero intercept indicating bias in IVW estimates (32). MR-PRESSO identified horizontal pleiotropy through a global test and, if necessary, corrected potential pleiotropy by removing outliers (33). Additionally, we implemented the “leave-one-out” method to exclude abnormal SNPs, preventing undue influence of individual SNPs on the causal relationship between exposure and outcome. Furthermore, heterogeneity assessment used the Cochran Q test, and a P-value below 0.05 indicated present heterogeneity. In such cases, we employed the IVW random-effects model to estimate causal effects. In the absence of heterogeneity, the IVW fixed-effects model was considered the result of the IVW method. Moreover, considering multiple MR analyses between different exposures and a single outcome, we controlled for potential false-positive results due to multiple hypothesis testing using the false discovery rate (FDR) (34). Finally, we visually represented the results through forest plots, funnel plots, scatter plots, and “leave-one-out” plots.

## 3 Results

### 3.1 Exploration of the causal effect of immune cell phenotypes on AR

**Figure 1:**
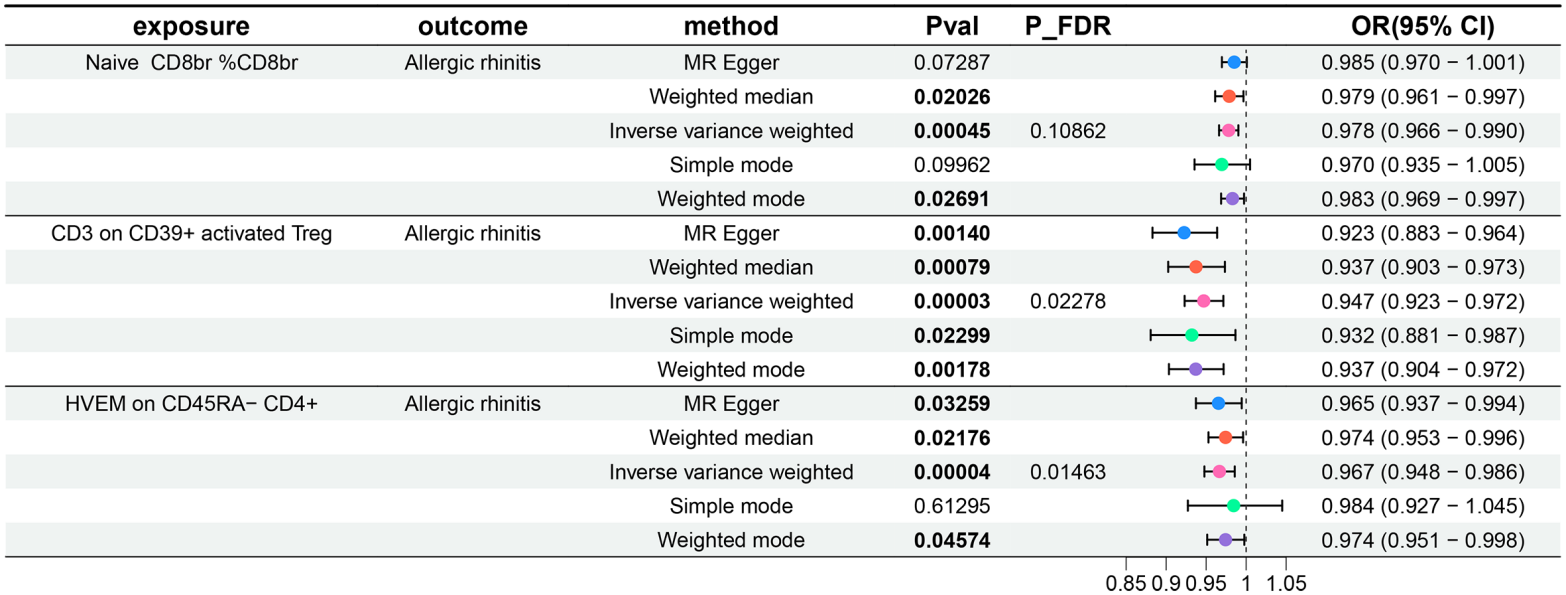
Forest plots show the causal effect of three immune cell phenotypes on AR. OR, odds ratio; CI, confidence interval.

**Figure 2:**
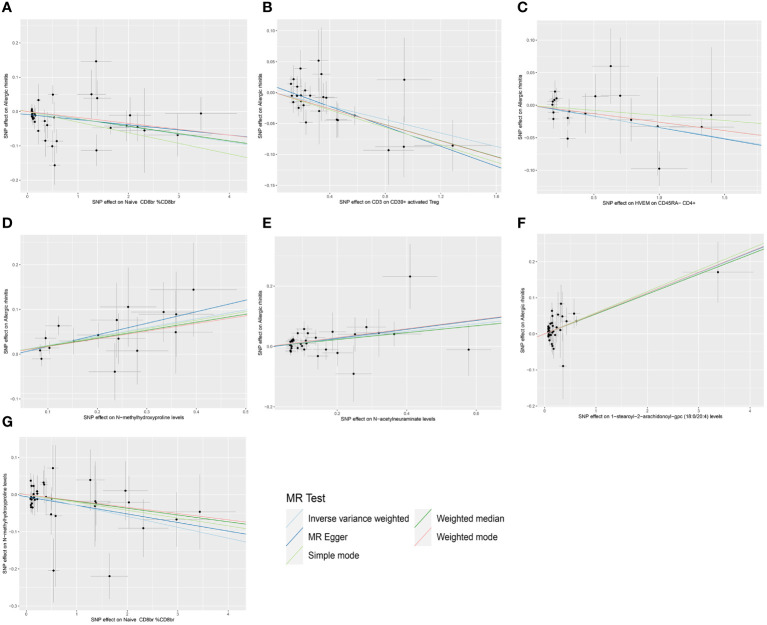
Scatter plots of MR analysis. **(A)** The causal effect of Naive CD8br % CD8br on AR. **(B)** The causal effect of CD3 on CD39+ activated Treg on AR. **(C)** The causal effect of HVEM on CD45RA- CD4+ on AR. **(D)** The causal effect of N-methylhydroxyproline levels on AR. **(E)** The causal effect of N-acetylneuraminate levels on AR. **(F)** The causal effect of 1-stearoyl-2-arachidonoyl-gpc (18:0/20:4) levels on AR. **(G)** The causal effect of Naive CD8br % CD8br on N-methylhydroxyproline levels.

For the three GWAS summary-level datasets mentioned above, we selected IVs based on established significance threshold levels and included them in this study. Their F-statistics were all above 10, indicating that weak instrument bias is unlikely to be significant (**Supplementary Tables 1–3, Sheet 1**). Employing the IVW method as the primary MR analysis, we conducted a two-sample MR analysis investigating the relationship between immune cell phenotypes and AR, accompanied by tests for heterogeneity and pleiotropy. Post multiple testing correction using the FDR method, we identified two immune cell phenotypes demonstrating a causal relationship with AR at a significance level of 0.05, both exerting protective effects: CD3 on CD39+ activated Treg (Treg panel) and HVEM on CD45RA- CD4+ (Maturation stages of T cell). The odds ratio (OR) of CD3 on CD39+ activated Treg against AR, calculated by the IVW method, was 0.947 (95%CI = 0.923–0.972, P = 3×10^–5^, P_FDR_ = 0.02278), and the OR of HVEM on CD45RA- CD4+ against AR, calculated by IVW, was 0.967 (95%CI = 0.948–0.986, P = 4×10^–5^, P_FDR_ = 0.01463). Additionally, following insights from previous research, a P_FDR_<0.2 was considered suggestive correlation (35). At a significance level of 0.2, we identified a third immune cell phenotype with a causal relationship with AR, also displaying a protective effect: Naive CD8br %CD8br (Maturation stages of T cell panel). The OR of Naive CD8br %CD8br against AR, calculated by IVW, was 0.978 (95%CI = 0.966–0.990, P = 4.5×10^–4^, P_FDR_ = 0.10862). Results from the other four MR analysis methods for these three immune cell phenotypes and AR mirrored those of the IVW method, with OR values less than 1. MR-Egger and MR-PRESSO indicated no horizontal pleiotropy (P-values > 0.05, **Supplementary Table 1, Sheet 1**), confirming the reliability of the analysis results. Forest plots representing three immune cell phenotypes as exposure and AR as an outcome are illustrated in **Figure 1**. Scatter plots in **Figures 2A–C**, and **Supplementary Figures 1–3 A, B, C** offer comprehensive insights into funnel plots, ‘leave-one-out’ plots, and individual forest plots. Additionally, the results of five MR analyses for the above three immune cell phenotypes and AR are provided in the **Supplementary Table 1, Sheet 2**.

### 3.2 Exploration of the causal effect of AR on immune cell phenotypes

**Figure 3:**
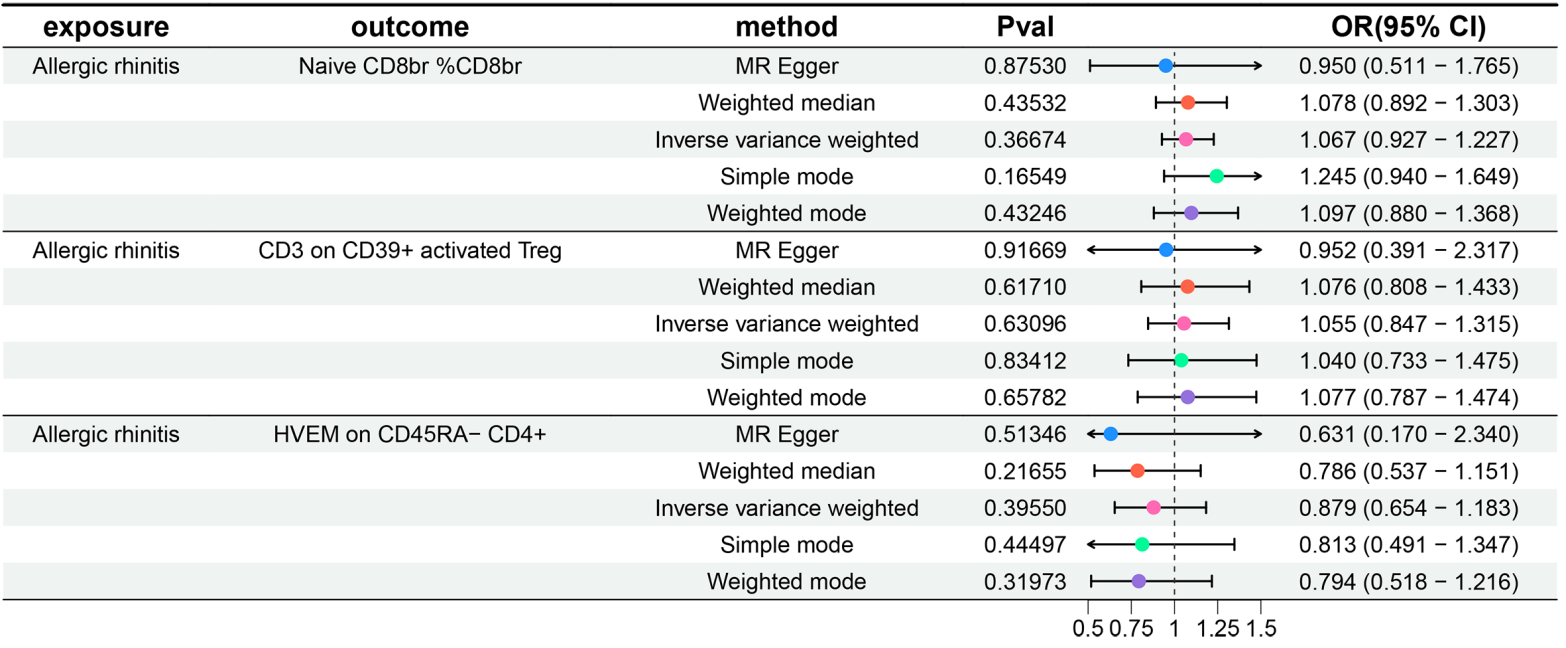
Forest plots show the causal effect of AR on three immune cell phenotypes. OR, odds ratio; CI, confidence interval.

Additionally, to meet the requirements of mediation MR studies, we further treated AR as the exposure and the three aforementioned immune cell phenotypes as outcomes, conducting a reverse MR analysis with IVW as the primary method. We did not observe any reverse causal relationships between the three immune cell phenotypes and AR (P-values all greater than 0.05). Details of the heterogeneity and pleiotropy tests are provided in the **Supplementary Table 2, Sheet 1**, the results of five MR analyses for AR and three immune cell phenotypes are provided in the **Supplementary Table 2, Sheet 2**, and the forest plots are illustrated in **Figure 3**.

### 3.3 Exploration of the causal effect of metabolite levels on AR

**Figure 4:**
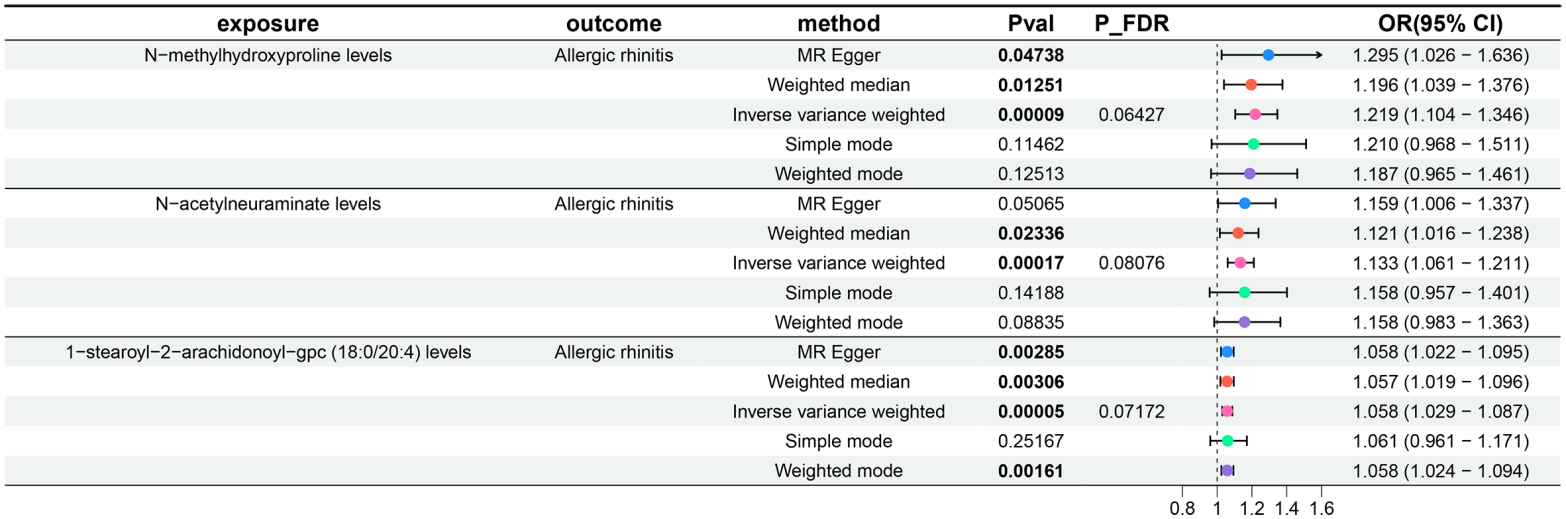
Forest plots show the causal effect of three metabolite levels on AR. OR, odds ratio; CI, confidence interval.

Subsequently, a two-sample MR analysis was conducted with metabolite levels as the exposure and AR as the outcome, utilizing IVW as the primary analytical method. Despite no significant associations at the 0.05 level after FDR correction for multiple testing, we identified three metabolite levels with causal relationships with AR at the 0.2 level, all acting as risk factors: N-methylhydroxyproline levels, N-acetylneuraminate levels, and 1-stearoyl-2-arachidonoyl-gpc (18:0/20:4) levels. The OR of N-methylhydroxyproline levels against AR, calculated by the IVW method, was 1.219 (95% CI = 1.104–1.346, P = 9×10^–5^, P_FDR_ = 0.06427). The OR of N-acetylneuraminate levels against AR, calculated by the IVW method, was 1.133 (95% CI = 1.061–1.211, P = 1.7×10^–4^, P_FDR_ = 0.08076). The OR of 1-stearoyl-2-arachidonoyl-gpc (18:0/20:4) levels against AR, calculated by the IVW method, was 1.058 (95% CI = 1.029–1.087, P = 5×10^–5^, P_FDR_ = 0.07172). Results from the other four methods in the MR analysis between these three metabolite levels and AR were consistent with the IVW method, with OR values all greater than 1. MR-Egger and MR-PRESSO indicated no horizontal pleiotropy (P-values all greater than 0.05, **Supplementary Table 3, Sheet 1**), confirming the reliability of the analysis results. Forest plots for these three metabolite levels as exposure and AR as the outcome are presented in **Figure 4**, scatter plots in **Figures 2D–F**), and **Supplementary Figures 1–3D–F** offer comprehensive insights into funnel plots, ‘leave-one-out’ plots, and individual forest plots. Furthermore, the detailed results for the five MR analyses between above three metabolite levels and AR can be found in the **Supplementary Table 3, Sheet 2**.

### 3.4 Exploration of the causal effect of immune cell phenotypes on metabolite levels

**Figure 5:**
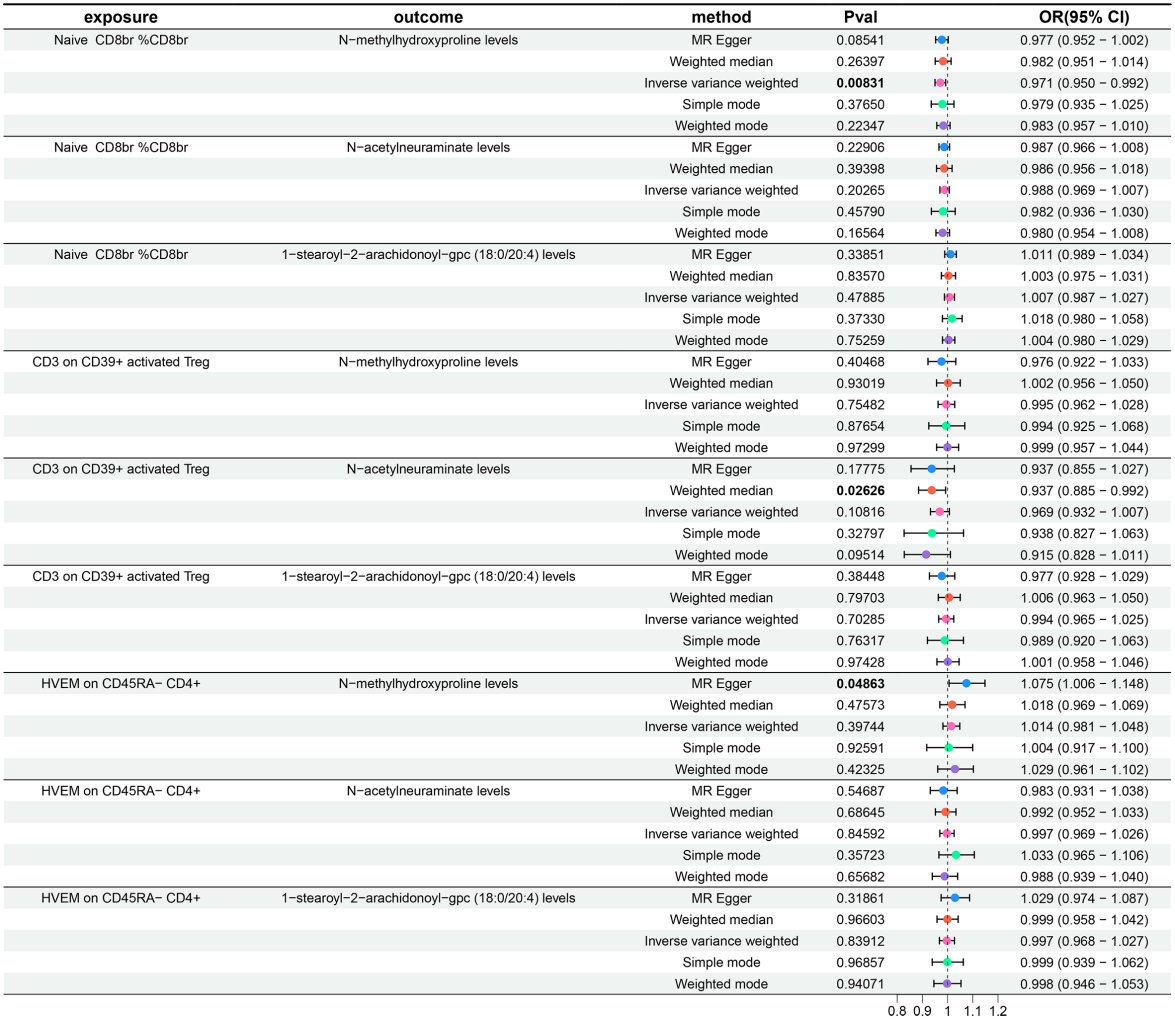
Forest plots show the causal effect of three immune cell phenotypes on three metabolite levels. OR, odds ratio; CI, confidence interval.

Following the identification of immune cell phenotypes and metabolite levels causally associated with AR, we conducted two-sample MR analyses between them. Since no reverse causal relationships were found in the second step of the MR analysis, the three immune cell phenotypes from the initial step met the criteria for mediation MR analysis. Due to the relatively small number of tests (only three tests between multiple exposures and a single outcome), we refrained from performing FDR correction in this segment of the study, considering a P-value less than 0.05 as statistically significant. Utilizing IVW as the primary analytical method, we established a causal relationship between the Naive CD8br %CD8br phenotype and N-methylhydroxyproline levels, indicating a protective effect. The OR of Naive CD8br %CD8br against N-methylhydroxyproline levels, calculated by the IVW method, was 0.971 (95% CI = 0.950–0.992, P = 8.31×10^–3^). OR values from all five MR analysis methods were consistently greater than 1, and both MR-Egger and MR-PRESSO indicated no horizontal pleiotropy (P-values all greater than 0.05, **Supplementary Table 4, Sheet 1**), affirming the robustness of the results. For the remaining eight MR analyses, given that the IVW method is the primary MR analysis method, we refrain from considering causal relationships between them, as their IVW method P-values were all greater than 0.05. The forest plots for these three immune cell phenotypes as exposure and three metabolite levels as the outcome are displayed in **Figure 5**, while **Figure 2G** illustrates the scatter plot for Naive CD8br %CD8br and N-methylhydroxyproline levels. **Supplementary Figures 1–3G** offer comprehensive insights into funnel plots, ‘leave-one-out’ plots, and individual forest plots. Moreover, the detailed results for the five MR analyses between three metabolite levels and three metabolite levels can be found in the **Supplementary Table 4, Sheet 2**.

### 3.5 Genetically predicted N-methylhydroxyproline levels mediate the association between Naive CD8+ T cells and AR

**Figure 6:**
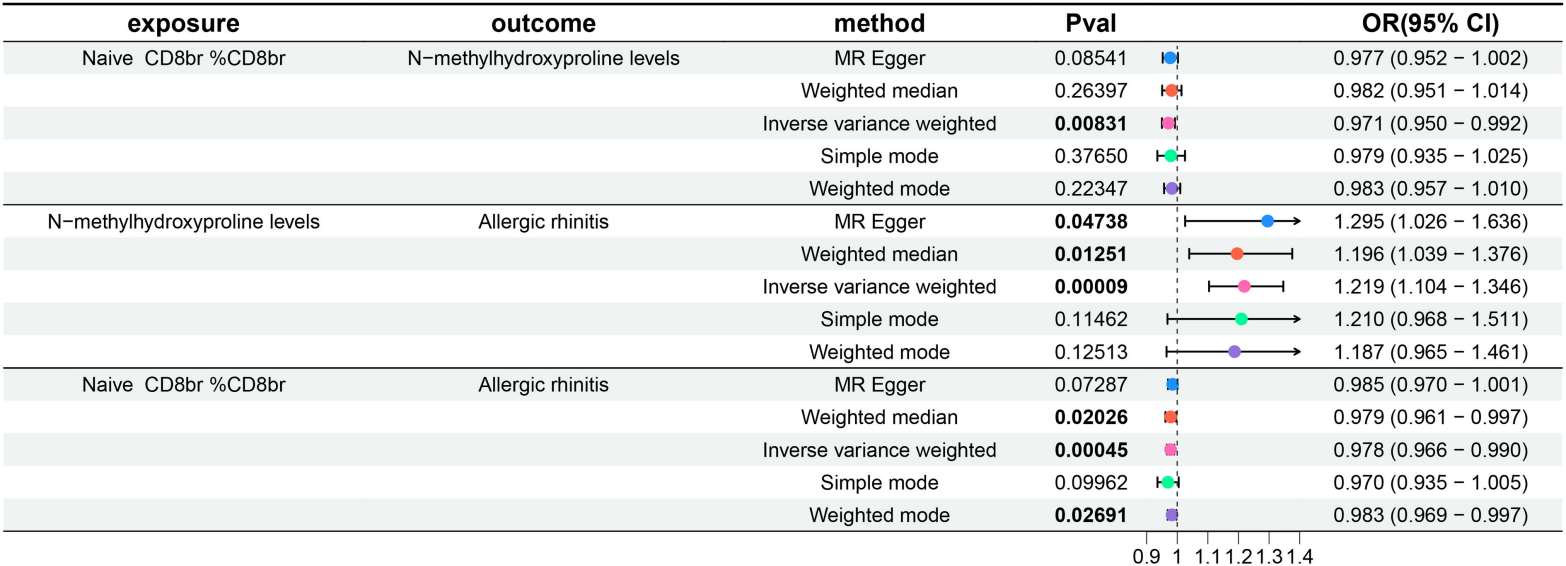
Forest plot of mediation MR analysis. OR, odds ratio; CI, confidence interval.

**Figure 7:**
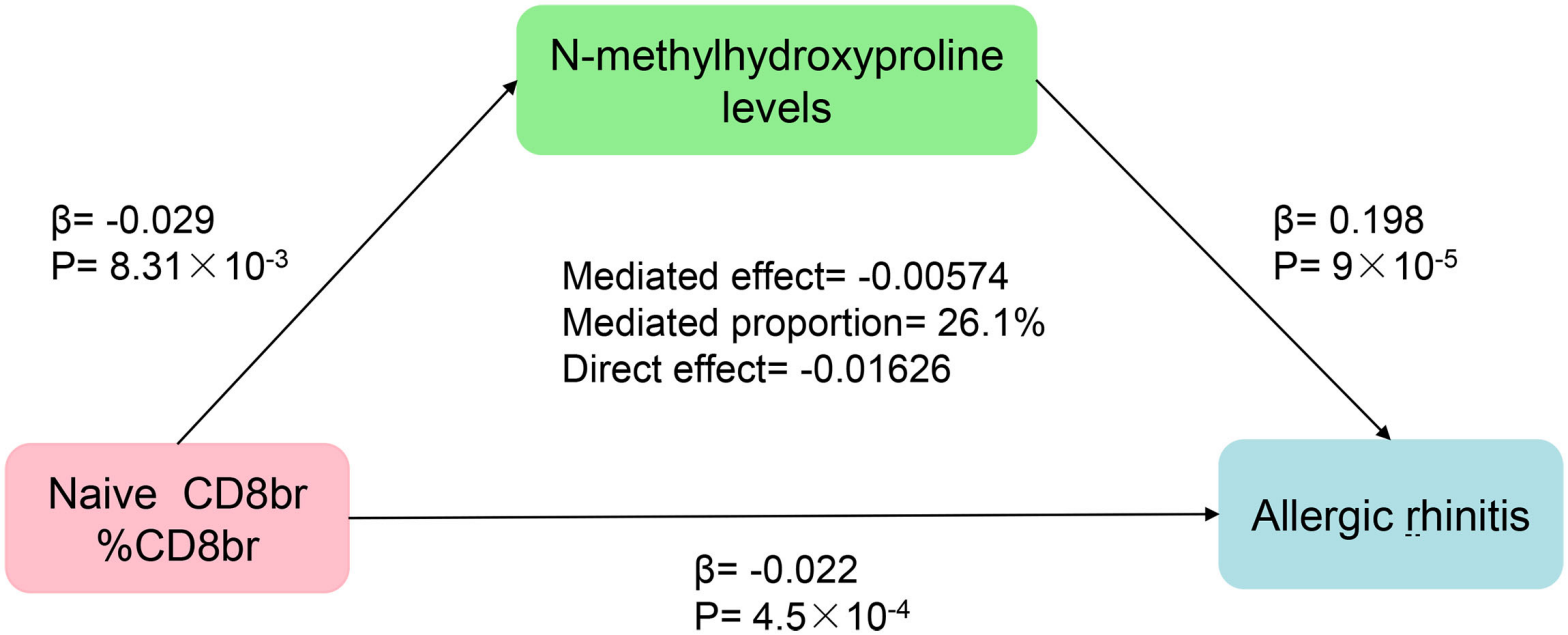
Schematic representation of the results from the mediation MR analysis.

Finally, the findings of our mediation MR analysis were summarized, and an intermediary factor was identified. **Figure 6** presents the summarized forest plot, while **Figure 7** depicts the schematic diagram of the mediation MR analysis. Notably, Naive CD8br %CD8br demonstrated a protective role in relation to N-methylhydroxyproline levels (β = -0.029, P = 8.31×10^–3^), with N-methylhydroxyproline levels identified as a risk factor for AR (β = 0.198, P = 9×10^–5^). Furthermore, Naive CD8+ %CD8+ emerged as a protective factor against AR (β = -0.022, P = 4.5×10^–4^). The mediated effect was -0.00574, accounting for 26.1% of the total effect, while the direct effect was -0.01626. Consequently, Naive CD8+ T cells contribute to the protection against AR by mitigating N-methylhydroxyproline levels.

## 4 Discussion

Utilizing publicly available GWAS summary-level data, we systematically explored the causal relationships between 731 immune cell phenotypes and AR. Furthermore, acknowledging the potential mediating influence of metabolites, we expanded our investigation to encompass the causal associations between 1400 metabolite levels and AR.

Previous studies have conducted MR analyses to investigate the causal relationship between eight immune-related diseases and AR. It indicates that atopic dermatitis (AD), asthma, and Crohn’s disease (CD) elevate the risk of AR, whereas Graves’ disease (GD) and systemic lupus erythematosus (SLE) potentially mitigate this risk (36). Another MR investigation scrutinized 486 circulating metabolites and 55 targeted urinary metabolites concerning their association with atopic dermatitis, allergic rhinitis, and asthma. Meta-analysis unveiled several circulating and urinary metabolites potentially linked to the onset and advancement of allergic diseases (37). Undoubtedly, the findings of these studies carry substantial implications for formulating preventive and therapeutic interventions for allergic rhinitis. Building upon prior research, this study employed GWAS summary-level data comprising 731 immune cell phenotypes and 1400 metabolite levels. Initially, it conducted separate MR analyses to identify immune cell phenotypes and metabolite levels potentially causally linked to allergic rhinitis. Subsequently, via mediation MR analysis, it investigated and uncovered a potential pathway, thus holding significant implications for guiding clinical practice.

To our knowledge, this study is the pioneering use of mediation MR analysis to investigate the causal links among immune phenotypes, metabolites, and AR. Following multiple testing correction of P-values using the FDR method, our findings indicate a total of three immune cell phenotypes acting as protective factors for AR, alongside three metabolite levels identified as risk factors for the condition. Notably, Naive CD8+ T Cells were discerned to exert a protective effect on AR by mitigating the levels of N-methylhydroxyproline. The STROBE-MR checklist pertaining to this study is available in **Supplementary Table 5**.

The human immune system comprises innate and adaptive components. Unlike the nonspecific actions of the innate immune system, the adaptive immune system involves T lymphocytes and B lymphocytes, acts with specificity, and induces immune memory, enabling rapid responses in subsequent encounters. T lymphocytes, vital in cellular immune responses, consist of two main subsets: CD4-positive (CD4+) T cells, known as helper T cells (Th), and CD8-positive (CD8+) T cells, known as cytotoxic T cells (CTL) (38). These cells play crucial roles in pathogen defense. Naive CD8+ T cells, stimulated by antigens, differentiate into effector and memory T cells, participating in respective immune processes (39). Furthermore, the existence of Naive CD8+ T cells empowers the organism to fend off novel, undetected infections and ailments (40). Our investigation identified a heightened ratio of Naive CD8+ T cells to the overall CD8+ T cell count, potentially diminishing the susceptibility to AR. Naive CD8+ T cells constitute the reservoir of CD8+ T cells, having recently matured in the thymus without encountering antigens yet, and can promptly react, producing effector and memory T cells for immunological assaults upon exposure to the relevant allergen. Certain research has indicated that, under chronic stimulation or specific pathological conditions, such as chronic infections or cancer, CD8+ T cells may experience immune exhaustion, characterized by a gradual loss of function and activity in response to sustained stimuli (41). Regulatory CD8+ T cells have inhibitory effects during AR occurrences, impacting inflammatory responses (42–44). Though not primary participants in allergic reactions, CD8+ T cells may exhibit exhaustion in response to allergens. Thus, the reservoir of Naive CD8+ T cells might have a significant regulatory function in the immune response of AR patients. However, this represents a speculative hypothesis concerning the potential causes of chronic recurrent episodes in AR patients, and the precise mechanisms necessitate additional validation through clinical research.

The CD3 on CD39+ activated Treg refers to a distinct subset within CD4 regulatory T cells expressing both CD3 and CD39 on its surface. Our investigation demonstrates that an elevation in the CD3 on CD39+ activated Treg correlates with a decreased risk of AR. Treg, categorized within the CD4+ T cell subset, play a pivotal role in the immune response to AR. Study suggests that diverse Toll-like receptors play crucial immunomodulatory roles in the pathogenesis of AR (45, 46). Notably, TICAM-1, functioning as a vital adaptor protein in Toll-like receptor signaling domains, participates in the signal transduction of AR. Treg can exert significant immunomodulatory effects on AR through the TICAM-1 pathway (47). The findings of this study highlight that a specific cell subset within Treg may confer a protective effect against AR, holding substantial implications for guiding AIT strategies.

HVEM on CD45RA- CD4+ T cells denote a distinct subset of CD4+ T cells expressing HVEM on the cell surface while lacking CD45RA, belonging to the mature stage of T lymphocytes. Herpes virus entry mediator (HVEM) belongs to the tumor necrosis factor receptor superfamily (TNFRSF) and plays a pivotal role in immune response regulation, contributing to the preservation of mucosal immune homeostasis (48, 49). Several studies have underscored HVEM’s role in maintaining T cell homeostasis within the intestinal epithelium and safeguarding against invasion by enteropathogenic bacteria (50, 51). However, current clinical research on the involvement of HVEM in AR remains limited. CD45RA, a member of the CD45 antigen family, is a glycoprotein located on the surface of lymphocytes (52). Under specific antigenic stimulation, the expression of CD45RA on the surface of T cells is constrained, commonly regarded as an indicator of naive T cells (53). Consequently, HVEM on CD45RA- CD4+ T cells signifies mature CD4+ T cells expressing HVEM. Our study discloses that a subset of mature CD4+ T cells may serve as a protective factor in AR. This finding is anticipated to offer valuable guidance for future AIT.

N-methylhydroxyproline is a derivative of proline. Extensive research has demonstrated that proline metabolism plays a crucial role in diverse biological processes, including cell signal transduction, stress response, and energy generation. Additionally, it may contribute to the pathogenic mechanisms of microbial invaders. Consequently, inhibiting the proline metabolism process emerges as a potential therapeutic strategy against the intrusion of specific pathogens into the body (54). The involvement of proline metabolism in the immune response triggered by allergens remains unclear. This investigation brings to light that N-methylhydroxyproline may pose a risk factor for AR, suggesting a promising avenue for future exploration in AR research.

N-acetylneuraminate, also known as sialic acid, represents a neuraminic acid embellished with an acetyl group, typically positioned at the terminal end of polysaccharide chains (55). Current research underscores that diverse pathogenic bacteria can exploit sialic acid for camouflage, eluding detection by the host’s innate immune system, thus gaining entry to enact their pathogenic pathways. Moreover, sialic acid can function as a nutritional source for select pathogenic bacteria (56, 57). This study posits that N-acetylneuraminate may constitute a potential risk factor for AR. An elevation in N-acetylneuraminate metabolite levels could, to some extent, create a conducive environment for the infiltration of pathogenic bacteria. When certain pathogenic microorganisms act as allergens, they might instigate an immune response in the body, culminating in the onset of AR. Presently, there exists a research gap concerning the correlation between N-acetylneuraminate and AR, necessitating further clinical investigations to validate these findings.

1-stearoyl-2-arachidonoyl-gpc is a lipid molecule derived from the amalgamation of stearic acid and arachidonic acid, constituting a glycerophospholipid. This investigation postulates that 1-stearoyl-2-arachidonoyl-GPC may play a role in the onset of AR. Regretfully, there is presently a dearth of clinical research concerning this metabolite, necessitating subsequent exploration in the future.

Through mediation MR analysis, we have discerned that N-methylhydroxyproline levels mediate the correlation between Naive CD8+ T cells and AR. The protective impact of Naive CD8+ T cells on AR manifests through a reduction in the N-methylhydroxyproline levels. It is conjectured that Naive CD8+ T cells, following exposure to the corresponding allergen, may influence N-methylhydroxyproline through a specific pathway, thereby mitigating the risk of AR occurrence.

This study conducted MR analysis utilizing publicly available comprehensive GWAS summary-level data, incorporating a large sample size and employing diverse testing methods to mitigate potential biases, thus demonstrating considerable statistical robustness. However, the study does have several limitations. Firstly, the absence of individual information on AR patients precludes further subgroup analysis. Secondly, the primary focus of this study is to investigate the potential mediated effect of metabolites between immune cell phenotypes and AR. Consequently, the impact of AR on immune cell phenotypes was not comprehensively analyzed. Thirdly, MR studies hinge on genetic variations for causal inference; therefore, to authenticate our research findings, additional clinical studies remain imperative. Furthermore, our study may serve as a guiding framework for identifying and validating biomarkers as therapeutic targets for AR in clinical practice, introducing new perspectives to future AIT, and fostering clinical advancements.

## 5 Conclusion

Our MR analysis explored the causal relationships between several immune phenotypes and metabolite levels with AR, shedding light on several factors potentially implicated in the occurrence of AR. Our study has alleviated the influence of confounding factors in clinical research, bolstering the reliability of the study results. Furthermore, employing mediation MR analysis, we unveiled a potential pathway to prevent the onset of AR, providing novel research directions for AIT.

In conclusion, this study represents a pioneering exploration into the genetic perspective of AR pathogenesis, providing fresh insights for future treatment and prevention strategies. Building upon this research, it holds promise for innovative clinical interventions for AR patients. Nonetheless, further investigations are warranted to validate these findings and extend their applicability to broader populations.

## Data availability statement

The original contributions presented in the study are included in the article/**Supplementary Material**. Further inquiries can be directed to the corresponding author.

## Ethics statement

Ethical approval was not required for the study involving humans in accordance with the local legislation and institutional requirements. Written informed consent to participate in this study was not required from the participants or the participants’ legal guardians/next of kin in accordance with the national legislation and the institutional requirements.

## Author contributions

ZC: Methodology, Project administration, Software, Writing – original draft. YS: Data curation, Visualization, Writing – original draft. XD: Validation, Writing – original draft. XZ: Funding acquisition, Supervision, Writing – review & editing.
