# Supplementary figures and images for: Genetically predicted N-methylhydroxyproline levels mediate the association between naive CD8+ T cells and allergic rhinitis: a mediation Mendelian randomization study
Source: Front Immunol. 2024 May 23;15:1396246. doi: 10.3389/fimmu.2024.1396246 (PMC11153669; doi:10.3389/fimmu.2024.1396246)

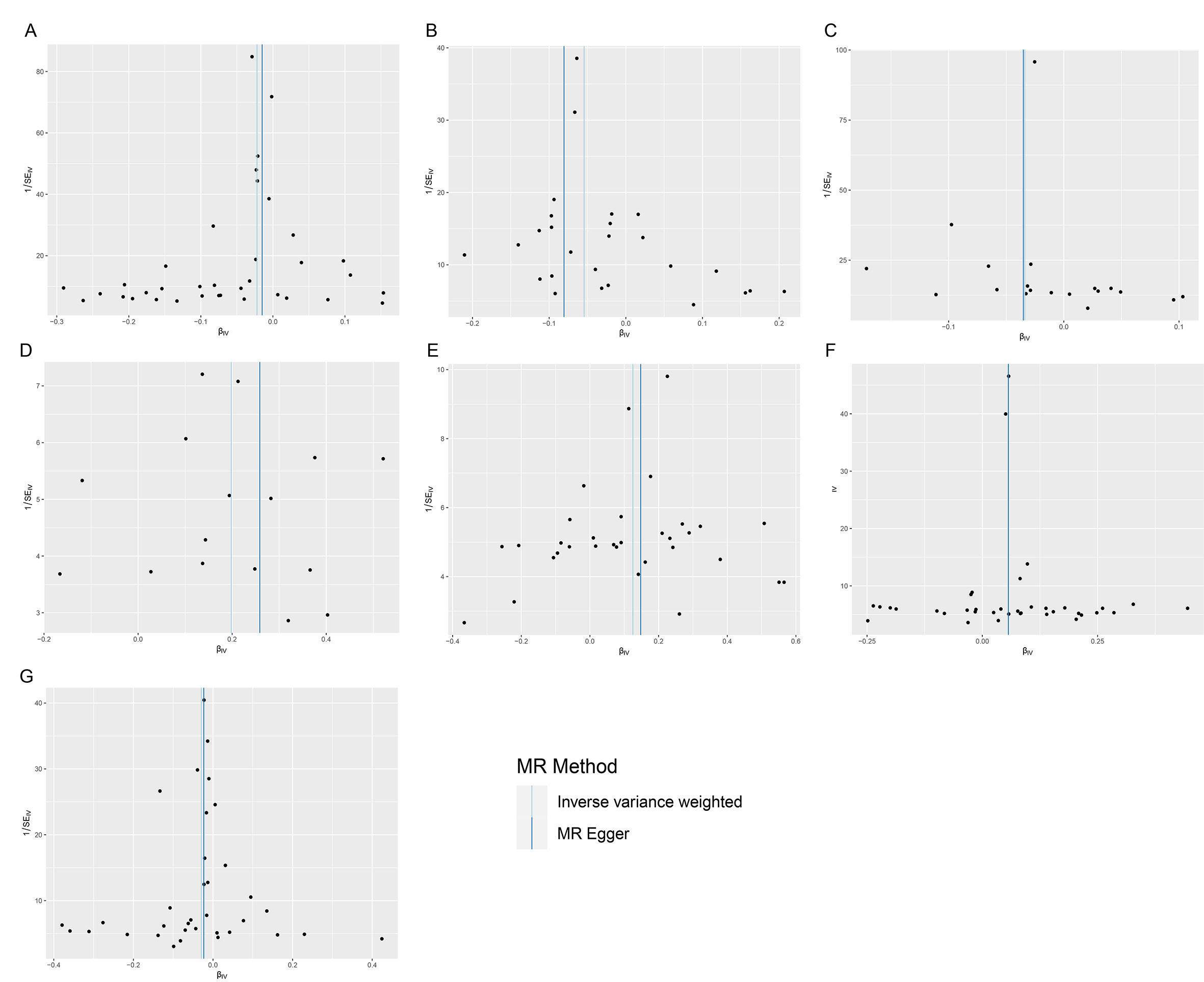

Supplement: Supplementary file 1 [file Image_1.tif]

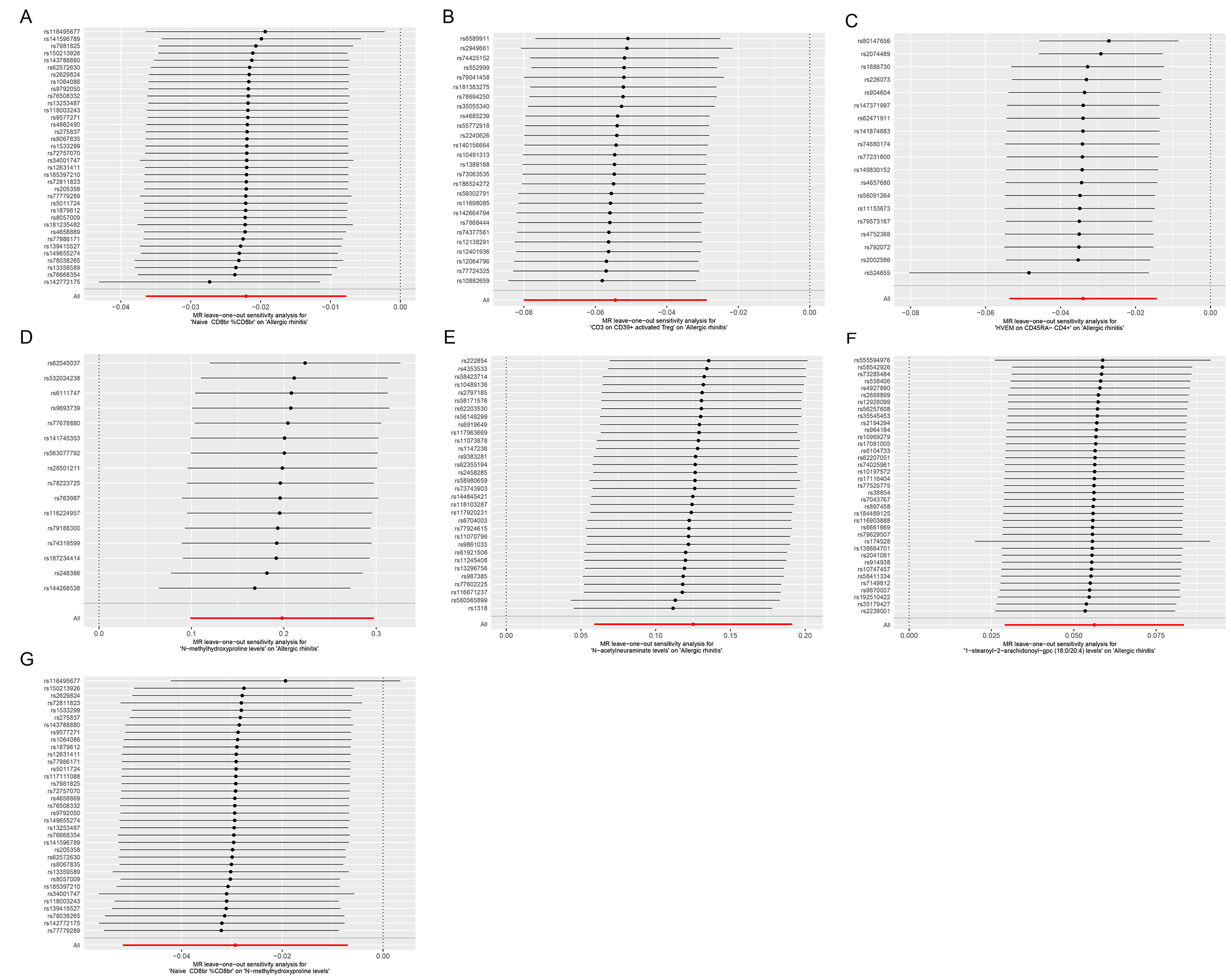

Supplement: Supplementary file 2 [file Image_2.tif]

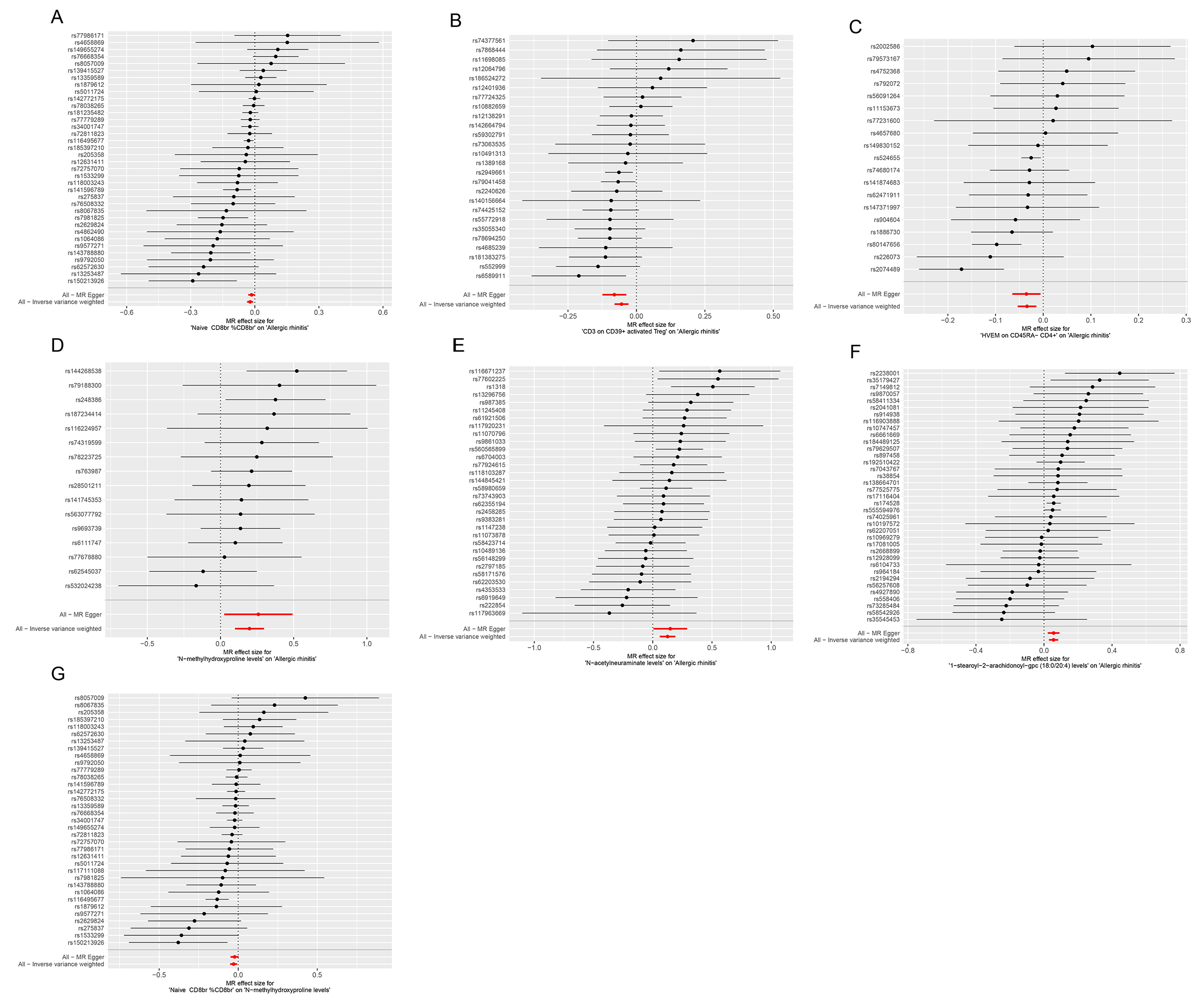

Supplement: Supplementary file 3 [file Image_3.tif]
