# Supplementary material for: Genetically predicted N-methylhydroxyproline levels mediate the association between naive CD8+ T cells and allergic rhinitis: a mediation Mendelian randomization study
Source: Front Immunol. 2024 May 23;15:1396246. doi: 10.3389/fimmu.2024.1396246 (PMC11153669; doi:10.3389/fimmu.2024.1396246)
Supplement: Supplementary file 8 [file Table_5.pdf]

Table 5 STROBE-MR checklist

STROBE-MR checklist of recommended items to address in reports of Mendelian randomization studies[1 2]

Item No.	Section		Checklist item	Relevant text from manuscript
1	**TITLE and ABSTRACT**		Indicate Mendelian randomization (MR) as the study's design in the title and/or the abstract if that is a main purpose of the study	Genetically predicted N-methylhydroxyproline levels mediate the association between naive CD8+ T cells and allergic rhinitis: a mediation Mendelian randomization study
	**INTRODUCTION**			
2	**Background**		Explain the scientific background and rationale for the reported study. What is the exposure? Is a potential causal relationship between exposure and outcome plausible? Justify why MR is a helpful method to address the study question	In recent years, MR has gained popularity......confounding factors and reverse causation (22).
3	**Objectives**		State specific objectives clearly, including pre-specified causal hypotheses (if any). State that MR is a method that, under specific assumptions, intends to estimate causal effects	Employing a two-sample MR approach......eliminate reverse causation.
	**METHODS**			
4	**Study design and data sources**		Present key elements of the study design early in the article. Consider including a table listing sources of data for all phases of the study. For each data source contributing to the analysis, describe the following:	
		a)	Setting: Describe the study design and the underlying population, if possible. Describe the setting, locations, and relevant dates, including periods of recruitment, exposure, follow-up, and data collection, when available.	Summary-level data regarding AR were extracted......under identifiers (GCST90199621 to GCST90201020).
		b)	Participants: Give the eligibility criteria, and the sources and methods of selection of participants. Report the sample size, and whether any power or sample size calculations were carried out prior to the main analysis	Summary-level data regarding AR were extracted......under identifiers (GCST90199621 to GCST90201020).
		c)	Describe measurement, quality control and selection of genetic variants	In MR analysis......between genetic variation and exposure (31).
		d)	For each exposure, outcome, and other relevant variables, describe methods of assessment and diagnostic criteria for diseases	The diagnosis of AR relied......and ICD-9 (477).
		e)	Provide details of ethics committee approval and participant informed consent, if relevant	Furthermore, the GWAS summary-level data used......considered not required.

5	**Assumptions**		Explicitly state the three core IV assumptions for the main analysis (relevance, independence and exclusion restriction) as well assumptions for any additional or sensitivity analysis	It is essential to clarify that MR......the outcome through exposure (24).
6	**Statistical methods: main analysis**		Describe statistical methods and statistics used	
		a)	Describe how quantitative variables were handled in the analyses (i.e., scale, units, model)	Employing five MR analysis methods......below 0.05 indicated present heterogeneity.
		b)	Describe how genetic variants were handled in the analyses and, if applicable, how their weights were selected	In MR analysis......between genetic variation and exposure (31).
		c)	Describe the MR estimator (e.g. two-stage least squares, Wald ratio) and related statistics. Detail the included covariates and, in case of two-sample MR, whether the same covariate set was used for adjustment in the two samples	Employing five MR analysis methods......considered the result of the IVW method.
		d)	Explain how missing data were addressed	To ensure robustness......SNPs on the causal relationship between exposure and outcome.
		e)	If applicable, indicate how multiple testing was addressed	Moreover, considering multiple MR......the false discovery rate (FDR) (34).
7	**Assessment of assumptions**		Describe any methods or prior knowledge used to assess the assumptions or justify their validity	Finally, for each SNP meeting the specified criteria......potential pleiotropy by removing outliers (33).
8	**Sensitivity analyses and additional analyses**		Describe any sensitivity analyses or additional analyses performed (e.g. comparison of effect estimates from different approaches, independent replication, bias analytic techniques, validation of instruments, simulations)	To ensure robustness......the result of the IVW method.
9	**Software and pre-registration**			
		a)	Name statistical software and package(s), including version and settings used	Data analyses were conducted......R software (version 4.3.1).
		b)	State whether the study protocol and details were pre-registered (as well as when and where)	The study was not pre-registered on any platforms.
	**RESULTS**			
10	**Descriptive data**			
		a)	Report the numbers of individuals at each stage of included studies and reasons for exclusion. Consider use of a flow diagram	Summary-level data regarding AR......under identifiers (GCST90199621 to GCST90201020).

		b)	Report summary statistics for phenotypic exposure(s), outcome(s), and other relevant variables (e.g. means, SDs, proportions)	Summary-level data regarding AR......under identifiers (GCST90199621 to GCST90201020).
		c)	If the data sources include meta-analyses of previous studies, provide the assessments of heterogeneity across these studies	Not provided in original research.
		d)	For two-sample MR:<br>i. Provide justification of the similarity of the genetic variant-exposure associations between the exposure and outcome samples<br>ii. Provide information on the number of individuals who overlap between the exposure and outcome studies	Significantly, there was no observed overlap, as samples of AR, immune cells, and metabolites originated from distinct consortia.
11	**Main results**			
		a)	Report the associations between genetic variant and exposure, and between genetic variant and outcome, preferably on an interpretable scale	Supplementary Table 1-4
		b)	Report MR estimates of the relationship between exposure and outcome, and the measures of uncertainty from the MR analysis, on an interpretable scale, such as odds ratio or relative risk per SD difference	Employing the IVW method as the primary MR analysis......protection against AR by mitigating N-methylhydroxyproline levels.
		c)	If relevant, consider translating estimates of relative risk into absolute risk for a meaningful time period	No relevant.
		d)	Consider plots to visualize results (e.g. forest plot, scatterplot of associations between genetic variants and outcome versus between genetic variants and exposure)	Figures 1-6, and Supplementary Figures 1-3.
12	**Assessment of assumptions**			
		a)	Report the assessment of the validity of the assumptions	For the three GWAS summary-level datasets...... (Supplementary Tables 1-3, Sheet 1).
		b)	Report any additional statistics (e.g., assessments of heterogeneity across genetic variants, such as I^2, Q statistic or E-value)	Supplementary Table 1-4.
13	**Sensitivity analyses and additional analyses**			
		a)	Report any sensitivity analyses to assess the robustness of the main results to violations of the assumptions	Supplementary Table 1-4.
		b)	Report results from other sensitivity analyses or additional analyses	Supplementary Table 1-4

		c)	Report any assessment of direction of causal relationship (e.g., bidirectional MR)	Additionally, to meet the requirements......(P-values all greater than 0.05).
		d)	When relevant, report and compare with estimates from non-MR analyses	No relevant.
		e)	Consider additional plots to visualize results (e.g., leave-one-out analyses)	Figures 1-6, and Supplementary Figures 1-3.
	**DISCUSSION**			
14	**Key results**		Summarize key results with reference to study objectives	Following multiple testing correction......levels of N-methylhydroxyproline.
15	**Limitations**		Discuss limitations of the study, taking into account the validity of the IV assumptions, other sources of potential bias, and imprecision. Discuss both direction and magnitude of any potential bias and any efforts to address them	This study conducted MR analysis......clinical studies remain imperative.
16	**Interpretation**			
		a)	Meaning: Give a cautious overall interpretation of results in the context of their limitations and in comparison with other studies	Through mediation MR analysis......thereby mitigating the risk of AR occurrence.
		b)	Mechanism: Discuss underlying biological mechanisms that could drive a potential causal relationship between the investigated exposure and the outcome, and whether the gene-environment equivalence assumption is reasonable. Use causal language carefully, clarifying that IV estimates may provide causal effects only under certain assumptions	The human immune system comprises......exploration in the future.
		c)	Clinical relevance: Discuss whether the results have clinical or public policy relevance, and to what extent they inform effect sizes of possible interventions	The human immune system comprises......exploration in the future.
17	**Generalizability**		Discuss the generalizability of the study results (a) to other populations, (b) across other exposure periods/timings, and (c) across other levels of exposure	Firstly, the absence of individual information......additional clinical studies remain imperative.
	**OTHER INFORMATION**			
18	**Funding**		Describe sources of funding and the role of funders in the present study and, if applicable, sources of funding for the databases and original study or studies on which the present study is based	This work was funded by grants from National Clinical Key Specialty Construction Project, Tianjin Key Medical Discipline (Specialty) Construction Project (TJYXZDXK-049A).
19	**Data and data sharing**		Provide the data used to perform all analyses or report where and how the data can be accessed, and reference these sources in the article. Provide the statistical code needed to reproduce the results in the article, or report whether the code is publicly accessible and if so, where	The original contributions presented......the corresponding author.
20	**Conflicts of Interest**		All authors should declare all potential conflicts of interest	The authors declare that...... potential conflict of interest.

This checklist is copyrighted by the Equator Network under the Creative Commons Attribution 3.0 Unported (CC BY 3.0) license.

1. Skrivankova VW, Richmond RC, Woolf BAR, Yarmolinsky J, Davies NM, Swanson SA, et al. Strengthening the Reporting of Observational Studies in Epidemiology using Mendelian Randomization (STROBE-MR) Statement. JAMA. 2021;under review.

2. Skrivankova VW, Richmond RC, Woolf BAR, Davies NM, Swanson SA, VanderWeele TJ, et al. Strengthening the Reporting of Observational Studies in Epidemiology using Mendelian Randomisation (STROBE-MR): Explanation and Elaboration. BMJ. 2021;375:n2233.
